# Leukocyte differential gene expression prognostic value for high versus low seizure frequency in temporal lobe epilepsy

**DOI:** 10.1186/s12883-023-03459-1

**Published:** 2024-01-02

**Authors:** Ryan Sprissler, Michael Hammer, David Labiner, Neil Joshi, Albert Alan, Martin Weinand

**Affiliations:** 1 Center for Applied Genetics and Genomic Medicine, RII, University of Arizona, Tucson, AZ USA; 2 Department of Neurology, University of Arizona College of Medicine, Tucson, AZ USA; 3 Department of Neurosurgery, University of Arizona College of Medicine, Tucson, AZ USA; 4 University of Arizona College of Medicine, Tucson, AZ USA

**Keywords:** Leukocyte, Gene expression, Temporal lobe epilepsy, Seizure frequency

## Abstract

**Background:**

This study was performed to test the hypothesis that systemic leukocyte gene expression has prognostic value differentiating low from high seizure frequency refractory temporal lobe epilepsy (TLE).

**Methods:**

A consecutive series of patients with refractory temporal lobe epilepsy was studied. Based on a median baseline seizure frequency of 2.0 seizures per month, low versus high seizure frequency was defined as ≤ 2 seizures/month and > 2 seizures/month, respectively. Systemic leukocyte gene expression was analyzed for prognostic value for TLE seizure frequency. All differentially expressed genes were analyzed, with Ingenuity® Pathway Analysis (IPA®) and Reactome, to identify leukocyte gene expression and biological pathways with prognostic value for seizure frequency.

**Results:**

There were ten males and six females with a mean age of 39.4 years (range: 16 to 62 years, standard error of mean: 3.6 years). There were five patients in the high and eleven patients in the low seizure frequency cohorts, respectively. Based on a threshold of twofold change (*p* < 0.001, FC > 2.0, FDR < 0.05) and expression within at least two pathways from both Reactome and Ingenuity® Pathway Analysis (IPA®), 13 differentially expressed leukocyte genes were identified which were all over-expressed in the low when compared to the high seizure frequency groups, including NCF2, HMOX1, RHOB, FCGR2A, PRKCD, RAC2, TLR1, CHP1, TNFRSF1A, IFNGR1, LYN, MYD88, and CASP1. Similar analysis identified four differentially expressed genes which were all over-expressed in the high when compared to the low seizure frequency groups, including AK1, F2R, GNB5, and TYMS.

**Conclusions:**

Low and high seizure frequency TLE are predicted by the respective upregulation and downregulation of specific leukocyte genes involved in canonical pathways of neuroinflammation, oxidative stress and lipid peroxidation, GABA (γ-aminobutyric acid) inhibition, and AMPA and NMDA receptor signaling. Furthermore, high seizure frequency-TLE is distinguished prognostically from low seizure frequency-TLE by differentially increased specific leukocyte gene expression involved in GABA inhibition and NMDA receptor signaling. High and low seizure frequency patients appear to represent two mechanistically different forms of temporal lobe epilepsy based on leukocyte gene expression.

## Background

Temporal lobe epilepsy (TLE) is the most common and medically refractory form of focal epilepsy in adults [1, 2]. Among patients with TLE, approximately two-thirds may be rendered seizure-free with anticonvulsant therapy [3]. For those patients in whom seizures persist despite maximum medical management, epilepsy surgery is a potentially curative treatment. Surgical treatment of TLE may include ablation or resection of temporal lobe epileptic tissue, including stereotactic laser amygdalohippocampotomy (SLAH) or amygdalohippocampectomy (AH) with or without anterior temporal lobectomy (ATL), respectively. In patients with medically refractory TLE, seizure-freedom may occur in approximately 60% of patients treated with ablative surgery (i.e. SLAH) and up to 80% of patients treated with resective surgery (i.e. AH with or without ATL) [4, 5].

Seizure frequency has traditionally served as a clinical measure of temporal lobe epilepsy severity (i.e. epileptogenicity) and as a quantitative measure of the TLE response to medical and surgical therapy [6]. In the emerging era of personalized medicine, the development of biomarkers may improve the quantitative assessment of the severity and response to treatment of various disease states [7]. Biomarkers of disease may offer insight into disease pathophysiology, potentially improving the development of novel therapies [7]. Biomarkers may predict the response of disease to medical or surgical treatment [7, 8]. One such biomarker of disease is leukocyte gene expression. Leukocyte gene expression may reflect central nervous system (CNS) disease severity, possibly through brain leukocyte trafficking [8, 9]. Systemic leukocyte gene expression may recapitulate the pathophysiology of CNS disease, indicate disease *diagnosis and severity* and predict *response* to therapy [8]. For example, down-regulation of leukocyte genes for mitochondrial protein synthesis, mitophagy, and stress defense is associated with the diagnosis of Parkinson’s Disease [10]. Leukocyte gene expression for B-cell receptor signaling and apoptosis correlates with the *severity* of depression [11]. In refractory temporal lobe epilepsy, leukocyte gene expression for lipid metabolism, oligodendrocyte morphology, inflammatory response, and astrocyte development has *predictive value* for seizure-freedom *response* to SLAH [8].

Temporal lobe epilepsy (TLE) in humans appears to manifest as a heterogeneous disorder, characterized by divergent functional neuroanatomical, microcellular, and signaling pathways that are contingent upon seizure frequency [12–14]. For instance, TLE patients with high seizure frequency exhibit heightened hippocampal to parahippocampal functional connectivity, whereas those with low seizure frequency demonstrate increased functional connectivity between the amygdala and parahippocampus [13]. Moreover, elevated seizure frequency in human TLE is correlated with a tendency towards enhanced neuronal loss, as indicated by diminished N-acetyl-aspartate (NAA) levels [14]. Additionally, high seizure frequency TLE, in comparison to low seizure frequency TLE, is characterized by more prominently deactivated signaling pathways [12]. In particular, the synaptoplastic deactivation of long-term depression (LTD) and dysregulation of long-term potentiation (LTP) disrupt the balance of synaptic strengthening and pruning, resulting in alterations predominantly observed in high seizure frequency TLE patients [12]. Conversely, low seizure frequency TLE is sustained by the upregulation of genes implicated in synaptic plasticity [12]. Consequently, disparate patterns of temporal lobe functional connectivity, neuronal density, and gene expression may serve to distinguish human TLE based on high and low seizure frequency. The identification of a biomarker capable of dichotomizing TLE according to a clinical measure of epileptogenicity could hold significant implications for elucidating fundamental pathophysiological differences and devising efficacious therapeutic strategies tailored to low and high seizure frequency subtypes.

The current study was performed to test the hypothesis that systemic leukocyte gene expression differentiates low from high TLE seizure frequency. The results show that low seizure frequency TLE is differentiated from high seizure frequency TLE based on leukocyte gene expression. Low and high seizure frequency TLE are predicted by the respective upregulation and downregulation of specific leukocyte genes involved in canonical pathways of neuroinflammation, oxidative stress and lipid peroxidation, GABA (γ-aminobutyric acid) inhibition, and AMPA (α- amino-3-hydroxy-5-methyl-4-isoxazolepropionic acid) and NMDA (N-Methyl- d-aspartate) receptor signaling. Furthermore, high seizure frequency-TLE is distinguished prognostically from low seizure frequency-TLE by differentially increased specific leukocyte gene expression involved in GABA (γ-aminobutyric acid) inhibition and NMDA (N-Methyl- d-aspartate) receptor signaling. We also explore what appears to be a consistent pattern of processes involving phagosome formation, CREB (cAMP response element-binding protein) signaling and autophagy being suppressed or enhanced in the leukocytes of high and low seizure frequency patients and how these two groups appear to represent two mechanistically different forms of TLE based on leukocyte gene expression.

## Methods

### Inclusion criteria

This study was conducted in accordance with the protocols and consent forms approved by the University of Arizona Institutional Review Board. The study recruited a diverse group of healthy pediatric and adult patients aged between 6 and 80 years who were referred for evaluation at the Arizona Comprehensive Epilepsy Program (ACEP), located at The University of Arizona in Tucson, AZ. The primary objective of the recruitment process was to characterize seizure phenomenology and determine the suitability of these patients for epilepsy surgery. Patients were recruited through both the Arizona Comprehensive Epilepsy Program and the neurosurgery Outpatient Clinic, ensuring a comprehensive selection process.

To be included in the study, patients had to have undergone a pre-surgical evaluation conducted by the ACEP to confirm the medical intractability of their condition. In addition, inclusion criteria required the identification of a seizure focus localized to a single temporal lobe. All selected patients were scheduled to undergo the standard surgical therapy for medically intractable temporal lobe epilepsy, following the well-established selection criteria set by the Case Management Conference of the University of Arizona Medical Center Arizona Comprehensive Epilepsy Program.

By implementing these rigorous inclusion criteria, we aimed to ensure that the study sample encompassed a diverse range of individuals across different age groups seeking evaluation and potential surgical intervention for epilepsy at ACEP. This approach allowed us to obtain comprehensive data on seizure phenomenology, evaluate the candidacy of patients for epilepsy surgery, and determine the most appropriate treatment strategies.

### Exclusion criteria

The participants recruited for this research study were subjected to a meticulous selection process, employing stringent exclusion criteria to uphold the study's integrity, safety, and cohort homogeneity. These exclusion criteria consisted of individuals with a prior history of epilepsy surgery on the brain and those with concurrent genetic disorders unrelated to epilepsy. Additionally, individuals with extra-temporal epilepsy, wherein seizures originate from regions other than the temporal lobe, were not considered for inclusion.

Furthermore, participants diagnosed with generalized or bitemporal lobar epilepsy were only included if the majority (at least 80%) of their seizures were found to originate from a single temporal lobe. Pregnant individuals and those undergoing anticoagulation or antiplatelet therapy that could not be safely reversed or temporarily discontinued were also excluded from the study. Moreover, individuals with unstable medical conditions such as unstable angina, poorly controlled diabetes, severe hypokalemia or hyponatremia, active infections that would hinder safe surgical therapy, and any medical condition determined by the Principal Investigator (the operating neurosurgeon) to be unsafe for neurosurgical intervention were not eligible for participation.

### Leukocyte gene expression and pathway analysis

In all sixteen patients, whole blood samples were obtained after all seizure focus localizing data confirmed a unilateral ictal temporal lobe seizure focus. Whole blood was stored in PaxGene RNA stabilization fluid (Qiagen, Valencia, CA) at -80 degrees centigrade. The technique for analyzing leukocyte gene expression was performed as previously described [8]. Briefly, using the RNeasy lipid tissue mini kit (Qiagen, Valencia, CA), total leukocyte RNA extraction was performed according to manufacturer!s directions. The SuperScript III kit (Life Technologies/Thermo Fisher Scientific, Carlsbad, CA) was used to produce first strand cDNA. The High Sensitivity RNA Analysis Kit (Fragment Analyzer; Advanced Analytical Technologies, Ankeny, IA) provided RNA quality assessment. Quant-iT RiboGreen RNA Assay Kit (Molecular Probes; Thermo Fisher Scientific, Carlsbad, CA) determined concentration of the isolated RNA. The stranded mRNA-Seq Kit (TDS KR0960 – v3.15; KapaBiosystems, Wilmington, MA) was used to construct RNASequence (RNA-Seq) Libraries. We determined average fragment size and quality by using the fragment Analyzer (Advanced Analytical Technologies, Ankeny, IA). We assessed library concentration using the Illumina Universal Adaptor-specific qPCR kit (KapaBiosystems, Wilmington, MA). Sequencing was performed on the HiSeq. 2500 (Illumina, San Diego, CA) with pooled and clustered equimolar samples and by using the Rapid-Run SBS 2 × 100 bp chemistry (Illumina, San Diego, CA) as previously described [15].

### Data analysis

Trimmomatic (USADelLab, Aachen, Germany) was used to trim and quality filter sample data. STAR aligner version 2.5.2b was used to align Fastq files against the GRCh37 reference genome [16] and Htseq-count version 0.6.1 was used to produce gene expression counts [17]. EdgeR’s exactTest function was used to calculate differential expression [8]. To eliminate composition biases between library samples, the calcNormFactors function in edgeR was used to normalize gene expression counts using the trimmed mean of M values (TMM) creating a set of scaling factors. All significant differentially expressed genes (FDR < 0.05) were analyzed with Ingenuity® Pathway Analysis (IPA®) to identify biological pathways with predictive value for low *versus* high temporal lobe epilepsy seizure frequency and for functional annotations wherein clustering of genes were significantly upregulated or downregulated (Qiagen, Hilden, Germany). IPA also predicted significant upstream transcriptional regulators. MDS plots were constructed using edgeR’s “plotMDS” function, which plots samples on a two-dimensional scatterplot so that distances on the plot approximate the typical log2 fold changes between samples. Heat maps were made using the R package heatmap version 1.0.12 implemented on the raw read count matrix.

## Results

### Patient characteristics

A total of sixteen patients participated in this study (Table 1). All sixteen patients met the Task Force of the ILAE (International League Against Epilepsy) Commission on Therapeutic Strategies definition of drug-resistant epilepsy [18]. All sixteen patients were determined by the University of Arizona Comprehensive Epilepsy Program to have intractable complex partial seizures originating from a single temporal lobe. Among the sixteen patients studied, there were ten males and six females with a mean age of 39.4 years (range: 16 to 62 years, standard error of mean: 3.6 years). The median baseline seizure frequency was 2.0 seizures per month (range: 0.25 to 60 seizures per month). There were five patients with high (> 2 seizures/month) and eleven patients with low (≤ 2 seizures/month) seizure frequency. The mean duration of temporal lobe epilepsy was 25.4 years (range: 4–61 years, standard error of mean: 4.3 years). There were eight patients with right- and eight patients with left-sided temporal lobe ictal seizure foci. The etiology of intractable temporal lobe epilepsy included stroke (*n* = 1), eclampsia (*n* = 1), traumatic brain injury (*n* = 1), infection (*n* = 1), abortion (*n* = 1), and unknown causes (*n* = 11) (Table 1). There were no statistically significant differences between the patients with high and low seizure frequency based on anticonvulsant medications taken (Table 2). There were no significant differences between the high seizure frequency (HSF) and low seizure frequency (LSF) groups on the basis of gender, age, ethnicity, duration of epilepsy, lateralization of temporal lobe epilepsy seizure foci, MRI brain evidence of medial temporal sclerosis, and concordance or dis-concordance of PET scan, neuropsychological testing, and ictal scalp or intracranial EEG data with definitive seizure focus localization (Table 3).

**Table 1:** Patient clinical demographics for temporal lobe epilepsy series

**Subject#**	**Gender**	**Age (yrs)**	**BSF (sz/mo)**	**Etiology**	**Duration (yrs)**	Laterality
1	M	38	3	Unk	17	R
2	M	37	0.25	Unk	35	L
3	M	60	0.25	Unk	47	L
4	M	26	1	Unk	4	R
5	F	32	0.33	CVA	8	R
6	M	16	4	Unk	10	R
7	F	35	1	Unk	13	R
8	F	54	1	Ecl	36	L
9	F	45	4	Abor	8	R
10	M	46	2	Unk	43	L
11	M	19	60	TBI	7	L
12	F	62	2	Inf	61	L
13	M	32	2	Unk	25	L
14	M	26	1	Unk	19	L
15	M	45	4	Unk	37	L
16	F	58	2	Unk	37	R

*Etiology* Etiology of epilepsy, *TBI* traumatic brain injury, *Unk* unknown, *CVA* stroke, *Abor* abortion, *Inf* infection, *Ecl* eclampsia, *Duration* duration of epilepsy, *Laterality* laterality of ictal temporal lobe seizure focus, *L* left, *R* right, *BSF* baseline seizure frequency, *sz/mo* seizures per month. Reproduced, in part, with permission of Sprissler et al. [8]

**Table 2:** Antiepileptic medication use of patients in temporal lobe epilepsy series

MEDICATION	USE (YES/NO)	LOW SEIZURE FREQUENCY	HIGH SEIZURE FREQUENCY	*P*-VALUE^#^
**CARBAMAZEPINE**	Yes	8	1	0.26
	No	4	3	
**PHENYTOIN**	Yes	6	3	0.58
	No	6	1	
**VALPROIC ACID**	Yes	4	2	1.00
	No	8	2	
**OXCARBAZEPINE**	Yes	3	1	1.00
	No	9	3	
**GABAPENTIN**	Yes	2	0	1.00
	No	10	4	
**TOPIRAMATE**	Yes	3	1	1.00
	No	9	3	
**PHENOBARBITAL**	Yes	3	1	1.00
	No	9	3	
**ZONISAMIDE**	Yes	2	0	1.00
	No	10	4	
**LEVETIRACETAM**	Yes	8	2	1.00
	No	4	2	
**VIGABATRIN**	Yes	0	1	0.25
	No	12	3	
**LACOSAMIDE**	Yes	1	1	1.00
	No	11	3	
**LAMOTRIGINE**	Yes	7	2	1.00
	No	5	2	
OTHER^*^	Yes	10	3	1.00
	No	2	1	

^*^Other = Lorazepam, Zomig, Clonazepam, Clobazam, Primidone, Fycompa,Temazepam, Mysoline, Diazepam; ^#^Fisher exact test. Reproduced, in part, with permission of Sprissler et al. [8]

**Table 3:** Demographic and seizure focus localization data of temporal lobe epilepsy series

DEMOGRAPHIC	FACTORS	HIGH SEIZURE FREQUENCY	LOW SEIZURE FREQUENCY	*P*-VALUE^*^
**GENDER**	Male	4	6	1.00
	Female	1	5	
**AGE (YEARS)**	Mean (SEM)	32.6 (6.3)	42.6 (4.2)	0.31^#^
**DURATION (YEARS)**	Mean (SEM)	15.8 (5.6)	29.8 (5.3)	0.17^#^
**ETHNICITY**	Caucasian	2	7	0.60
	Hispanic/Other	3	4	
**MRI BRAIN**	MTS	3	8	1.00
	Normal/Other	2	3	
**PET SCAN**	Concordant	2	9	0.18
	Dis-concordant	2	1	
**ICTAL SCALP EEG**	Concordant	4	11	0.31
	Dis-concordant	1	0	
**INTRACRANIAL EEG**	Localizing	3	3	1.00
	Non-localizing	0	0	
**NEUROPSYCH**	Concordant	3	3	0.55
	Dis-concordant	1	5	
**LATERALITY**	Left	2	6	1.00
	Right	3	5	

^*^Fisher exact test; ^#^Mann–Whitney U test. High & Low seizure frequency: > or ≤ 2/month, Concordant/Dis-concordant = Concordance or dis-concordance with temporal lobe ictal EEG seizure focus localization, MTS = medial temporal sclerosis, Duration = duration of epilepsy, Neuropsych = neuropsychological testing; Laterality = temporal lobe seizure focus lateralization (left versus right). Reproduced, in part, by permission of Sprissler et al. [8]

### Leukocyte differential gene expression associated with seizure frequency

Leukocyte differential gene expression analysis was performed comparing the high (*n* = 5) and low (*n* = 11) seizure frequency groups. Based on a threshold of twofold change (*p* < 0.001, FC > 2.0, FDR < 0.05) and the involvement in at least two biological pathways in both Reactome and Ingenuity® Pathway Analysis (IPA®), 13 differentially expressed genes (DEGs) were identified which were all overexpressed in the low when compared to the high seizure frequency groups (Table 4). Using the same filter criteria, four differentially expressed genes (DEGs) were identified which were all overexpressed in the high when compared to the low seizure frequency groups (Table 4).

**Table 4:** Leukocyte Differentially Expressed Genes (DEGs) comparing low and high temporal lobe epilepsy seizure frequency patients

Genes	Fold Change (FC)	FDR^a^	*p*-value
Upregulated in **Low** compared to **High** seizure frequency TLE			
NCF2	4.044962	0.002835	4.83 × 10^–6^
HMOX1	3.912648	0.001222	1.04 × 10^–6^
RHOB	3.370525	0.023331	0.000122
FCGR2A	3.355795	0.012951	4.92 × 10^–5^
PRKCD	3.068704	0.010221	3.32 × 10^–5^
RAC2	3.041648	0.034416	0.00029
TLR1	2.869047	0.035484	0.000348
CHP1	2.769877	0.008084	2.15 × 10^–5^
TNFRSF1A	2.732864	0.023331	0.00013
IFNGR1	2.732414	0.016528	6.99 × 10^–5^
LYN	2.525921	0.031583	0.00023
MYD88	2.520108	0.043643	0.000496
CASP1	2.443742	0.035484	0.00033
Upregulated in **High** compared to **Low** seizure frequency TLE			
TYMS	444.9515	0.000136	9.58 × 10^–9^
AK1	59.63923	0.009746	2.93 × 10^–5^
F2R	51.47103	0.015462	6.43 × 10^–5^
GNB5	22.58811	0.032269	2.62 × 10^–4^

*FC* indicates fold change of leukocyte gene upregulation in low versus high seizure frequency TLE. ^a^*FDR* False Discovery Rate. (*p* < 0.001, FC > 2.0, FDR < 0.05)

**Fig. 1:**
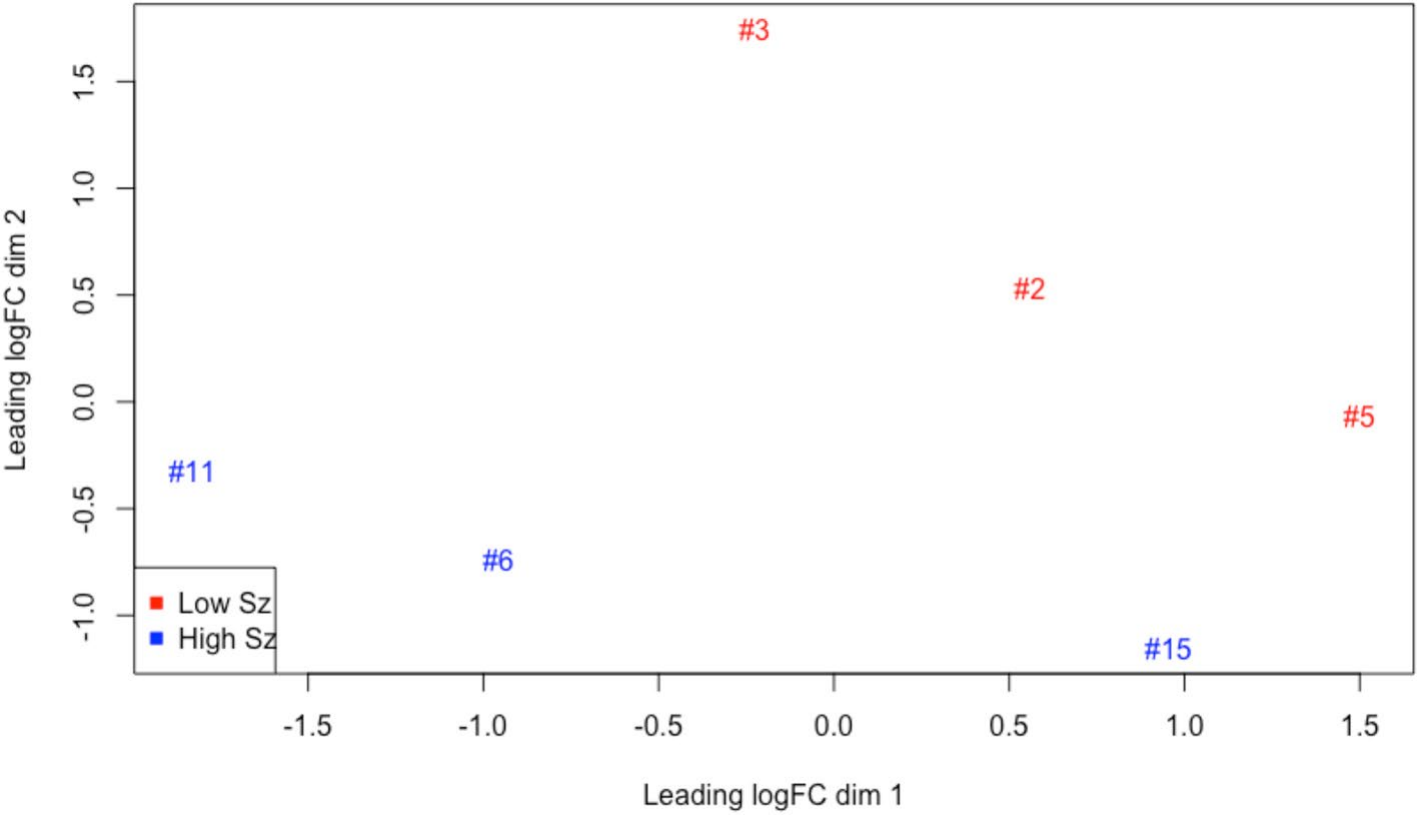
Multidimensional scaling plot (MDS) generated using edgeR showing segregation of the low and high seizure frequency patients’ leukocyte transcriptional profile. The three lowest and three highest seizure frequency samples were used to generate plot. Numbered sample IDs indicate patients from list in Table 1

**Fig. 2:**
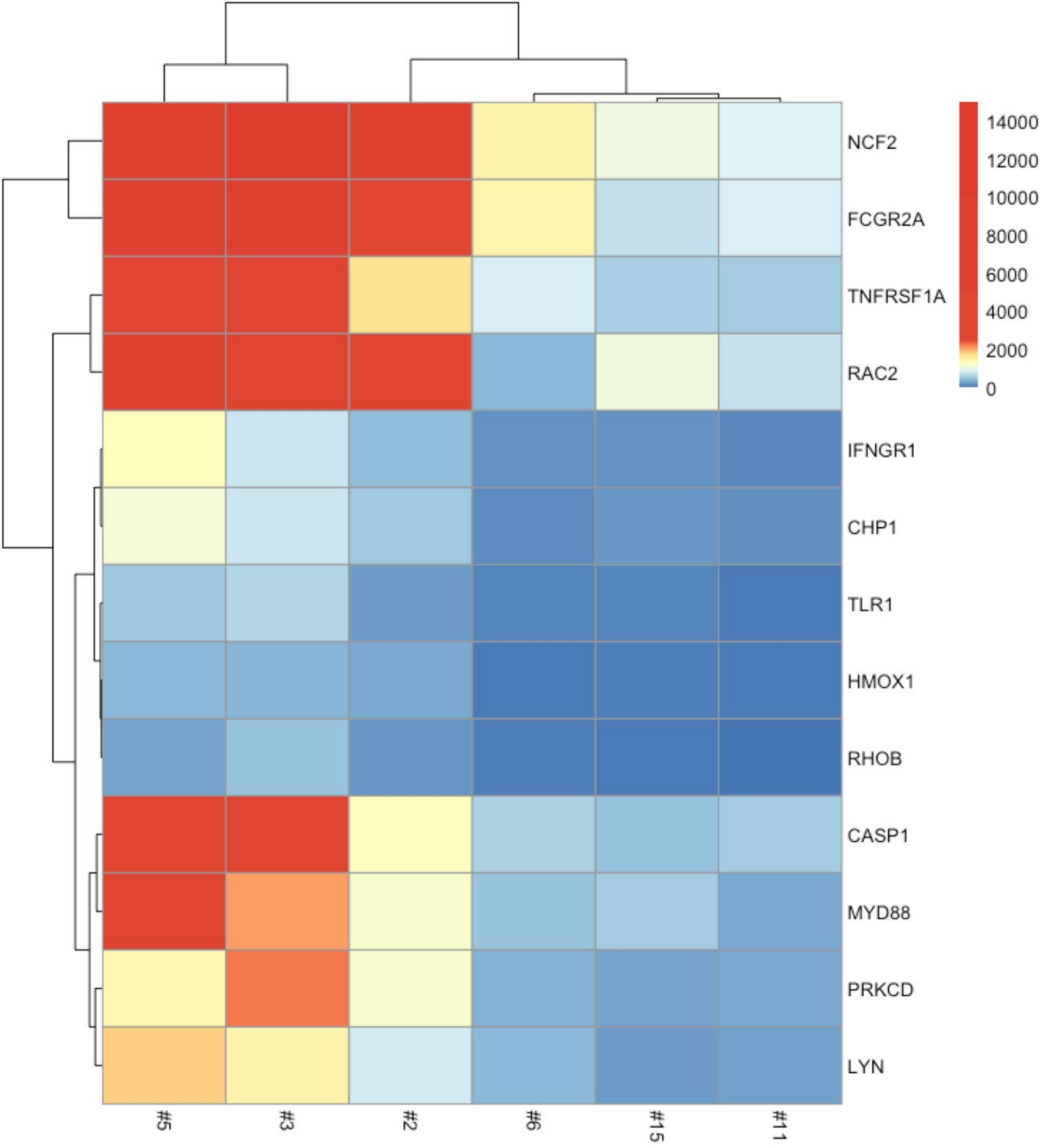
Heatmap generated in edgeR using the 13 most variable genes across samples. Unsupervised clustering showing the grouping of low (#5, #3, #2) and high (#6, #15, #11) seizure frequency patients. X-axis indicates sample IDs from subject list in Table 1. Red indicates a higher level of comparative expression while blue indicates a lower level of expression

To further illustrate the expression profile differences between the low and high seizure frequency groups, a multidimensional scaling plot (MDS) was generated using the three patients with the lowest seizure frequency and the three patients with the highest seizure frequency. This plot generated a clear division between these two cohorts indicating variable inter-group expression profiles (Fig. 1). Again using the three lowest and highest seizure frequency patients from the cohort, a heatmap was generated to assess the overall expression level of each identified DEG and classify as either a high or low expressing gene by actual quantity of transcripts within the sample (Fig. 2).

## Significantly activated biological pathways associated with seizure frequency

**Fig. 3:**
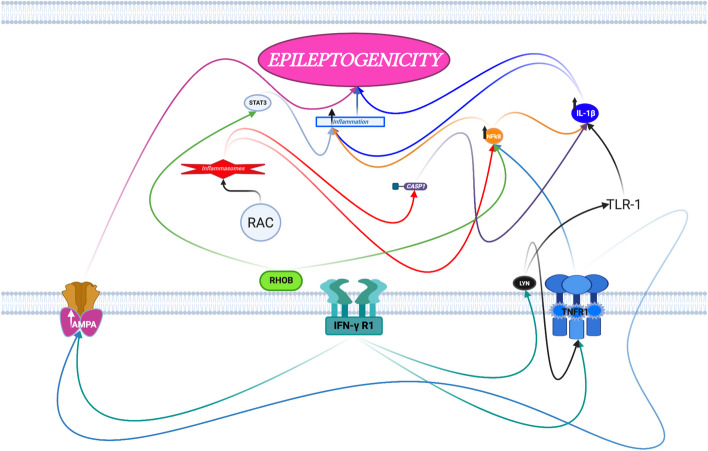
Leukocyte differentially expressed genes (DEGs) involved in neuroinflammation are upregulated in low but downregulated in high temporal lobe epilepsy seizure frequency. Figure prepared with BioRender.com

**Fig. 4:**
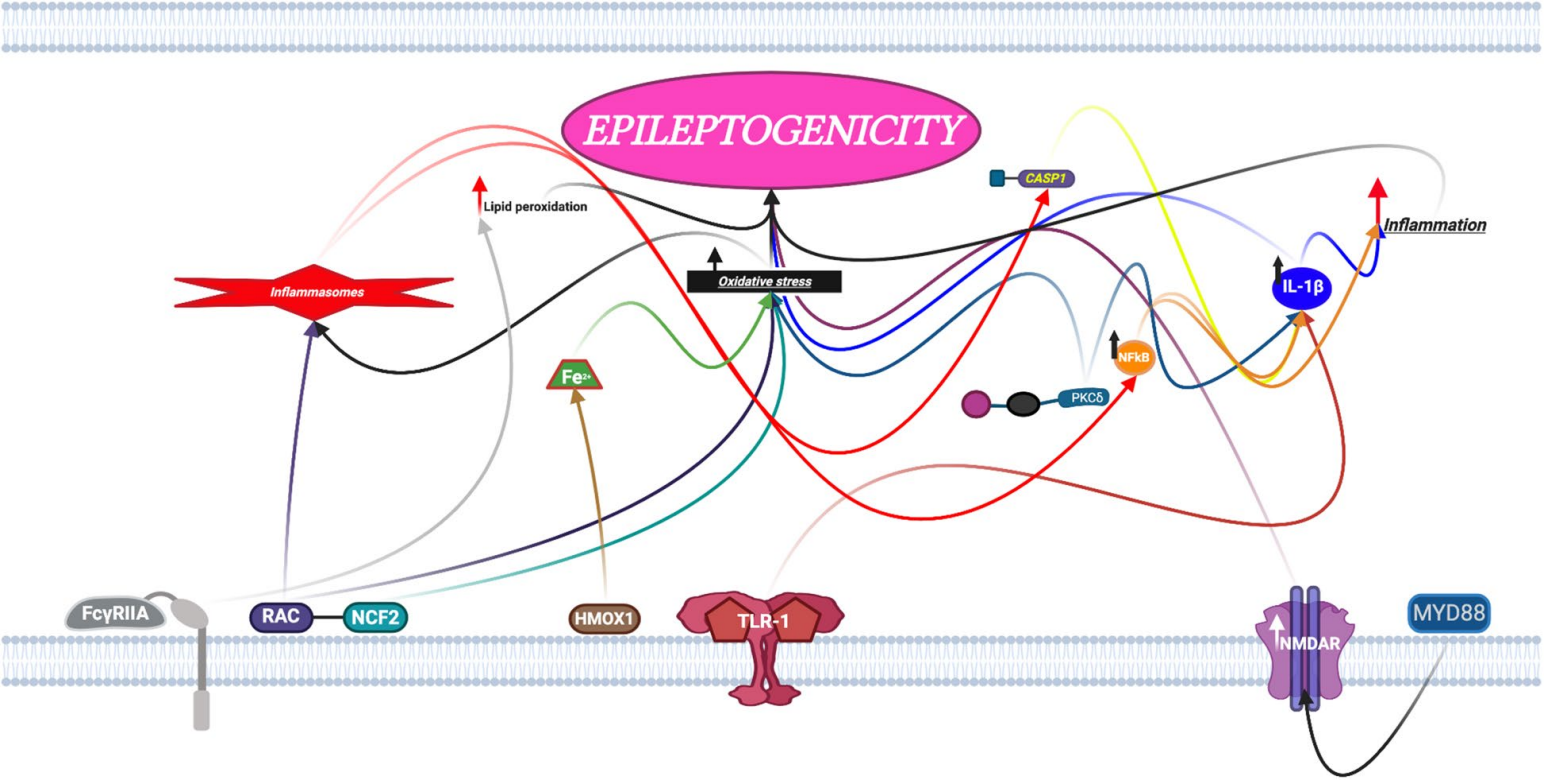
Leukocyte differentially expressed genes (DEGs) involved in oxidative stress and lipid peroxidation are upregulated in low but downregulated in high temporal lobe epilepsy seizure frequency. Figure prepared with BioRender.com

**Fig. 5:**
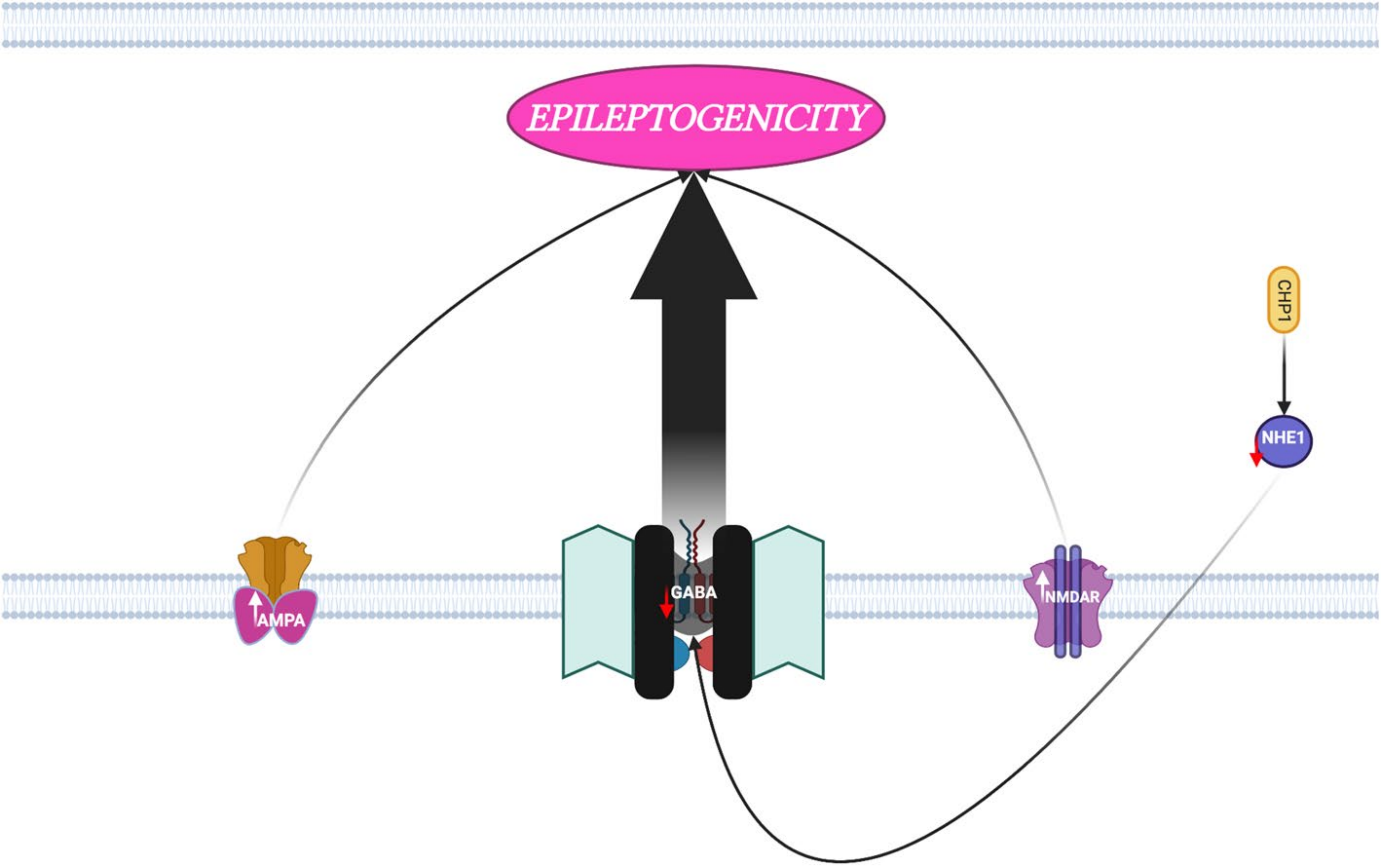
Leukocyte differentially expressed genes (DEGs) involved in glutamate/GABA-mediated excitotoxicity in temporal lobe epileptogenicity which are upregulated in low but downregulated in high temporal lobe epilepsy seizure frequency. Figure prepared with BioRender.com

Pathway analysis was performed on the above DEGs to detect significantly activated biological pathways in the low compared to the high seizure frequency group (*p* < 0.0001, z-score > 2.000) (Table 5). The results show that low human TLE seizure frequency is differentiated from high seizure frequency by the respective upregulation versus downregulation of specific leukocyte DEGs involving neuroinflammation, oxidative stress and lipid peroxidation, GABA (γ-aminobutyric acid) inhibition, and AMPA (α-amino-3-hydroxy-5-methyl-4- isoxazolepropionic acid) and NMDA (N-Methyl- d-aspartate) receptor signaling (Figs. 3, 4 and 5).

**Table 5:** Ingenuity® Pathway Analysis (IPA®) involving the DEGs with significant activation of biological pathways when comparing low to high seizure frequency groups

Biological Pathways	Genes	z-score	*p*-value
Upregulated in **Low** compared to **High** seizure frequency TLE			
Neuroinflammation Signaling Pathway	CASP1, CD86, CHP1, CX3CR1, FZD1, HMOX1, IFNGR1, MYD88, NCF2, SOD2, TLR1, TNFRSF1A	2.887	8.32 × 10^–7^
Fcγ Receptor-mediated Phagocytosis in Macrophages	FCGR2A, FCGR3A/FCGR3B, HMOX1, LYN,	2.646	2.0 × 10^–6^
and Monocytes	PRKCD, PTEN, RAC2		
Production of Nitric Oxide and Reactive Oxygen Species in	CAT, IFINGR1, LYZ, NCF2,	2.121	4.9 × 10^–6^
Macrophages	PRKCD, RAC2, RHOB,		
	SIRPA, TNFRSF1A		
Phospholipase C Signaling	ARHGEF11, CHP1, FCGR2A, GNB5, HMOX1,	2.449	5.6 × 10^–5^
	LYN, PRKCD, RAC2, RHOB		
Role of Pattern Recognition Receptors in Recognition of	C3AR1, C5AR1, CASP1,	2.449	7.9 × 10^–5^
Bacteria and Viruses	MYD88, NLRC4, PRKCD,		
	TLR1		
Upregulated in **High** compared to **Low** seizure frequency TLE			
Pyrimidine Deoxyribonucleotides De Novo Biosynthesis I^a^	AK1, TYMS		4.17 × 10^–4^
AMPK Signaling^a^	AK1, GNB5, PPM1K		4.47 × 10^–3^
Thrombin Signaling^a^	F2R, GNB5		3.39 × 10^–2^

Canonical Pathways with statistically significant p-values (*p* < 0.0001 and z-score > 2.000)

^a^indicates z-score unable to be calculated (NaN) for pathway by Ingenuity® Pathway Analysis (IPA®)

**Fig. 6:**
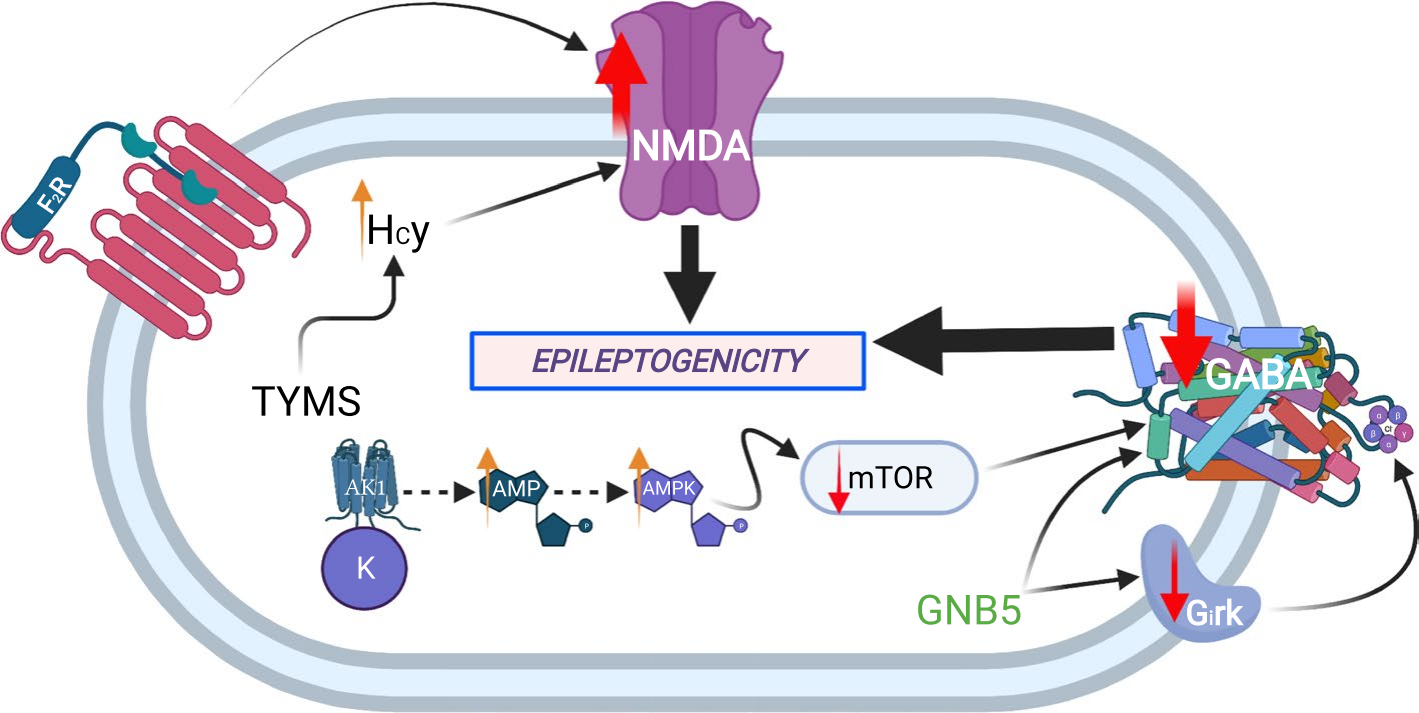
Leukocyte differentially expressed genes (DEGs) involved in NMDA facilitation and GABA inhibition which are upregulated in high compared to low temporal lobe epilepsy seizure frequency patients. Figure prepared with BioRender.com

**Fig. 7:**
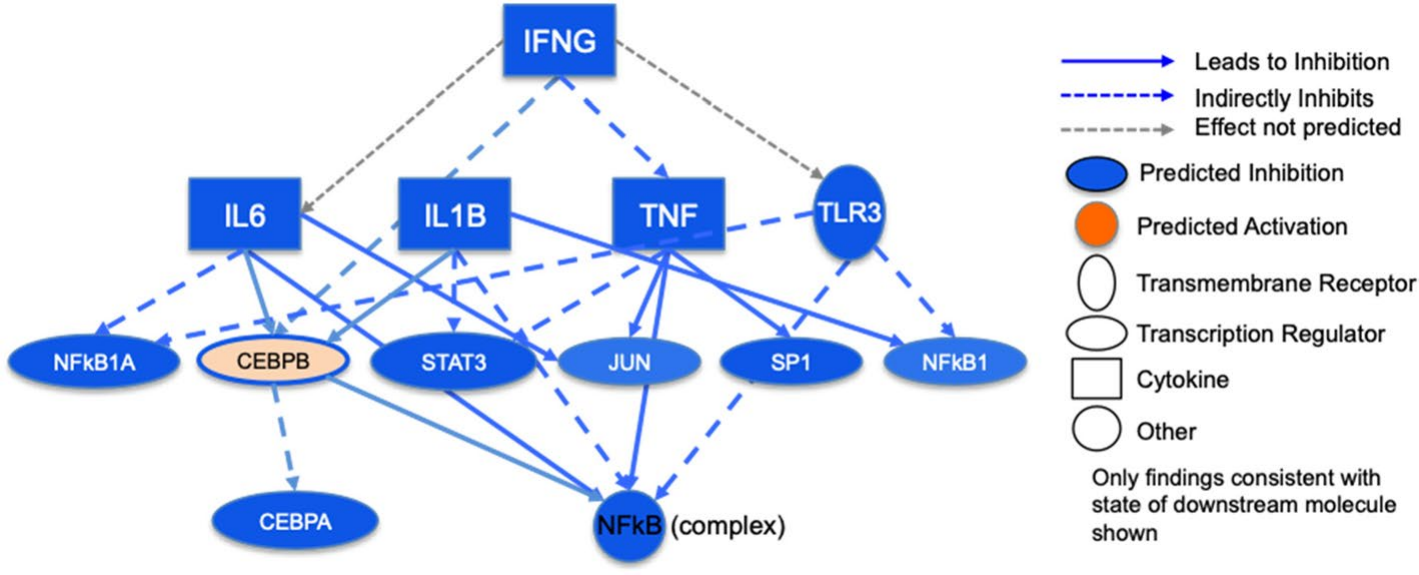
Upstream regulators predicted to drive the temporal lobe epilepsy response in leukocytes with an overlap *p*-value < 0.05. Blue cells indicate predicted inhibition of the pathway, orange shows predicted activation. The shades for colored cells represent z-scores values. Color of nodes indicates predicted activation (orange) or deactivation (blue). The NFKB complex is a key node in this network. Figure prepared using Ingenuity Pathway Analysis (IPA)

This same analysis was performed on the high compared to low DEGs to identify significantly activated biological pathways in the high compared to deactivated biological pathways in the low seizure frequency group (Table 5). These results indicate that high human TLE seizure frequency is differentiated from low seizure frequency by the respective upregulation versus downregulation of specific leukocyte DEGs involving NMDA receptor facilitation and GABAergic inhibition (Fig. 6). Additionally, the top altered canonical pathways were then inferred from pathway enrichment analysis. These include several related functions involved in phagosome formation, neuroinflammation signaling, CREB signaling, and autophagy showing a consistent pattern of suppression or enhancement in the leukocytes of HSF or LSF patients, respectively (Table 6). Predicted upstream transcriptional regulators can identify drivers of the differential expression in TLE. Of the biological molecules predicted to be inhibitory, IFNG was the regulator with the lowest p-value (4.94 × 10^–19^) and the most negative z-score (-4.71) (Fig. 7). Immunoglobulin was the top predicted upstream activator with a p-value of 3.10 × 10^–12^ and a z-score of 2.70 (data not shown).

**Table 6:** Top most significantly altered IPA canonical pathways

	*p*-value	z-score
1. Phagosome Formation	1.27 × 10^–8^	-4.03
2. Neuroinflammation Signaling Pathway	1.02 × 10^–7^	-2.89
3. CREB Signaling in Neurons	1.29 × 10^–6^	-2.84
4. Fcγ Receptor-mediated Phagocytosis in Macrophages and Monocytes	2.15 × 10^–6^	-2.65
5. Autophagy	6.17 × 10^–5^	-2.65
6. Production of Nitric Oxide and Reactive Oxygen Species in Macrophages	3.47 × 10^–5^	-2.12
7. fMLP Signaling in Neutrophils	1.95 × 10^–5^	-2.00
8. Role of Pattern Recognition Receptors in Recognition of Bacteria and Viruses	5.89 × 10^–5^	-2.45
9. CXCR4 Signaling	9.12. × 10^–5^	-2.24
10. TREM1 Signaling	1.26 × 10^–4^	-2.24
11. IL-8 Signaling	1.10 × 10^–4^	-2.45

## Discussion

The main finding of this study is that high and low seizure frequency TLE patients appear to represent two mechanistically different forms of temporal lobe epilepsy based on leukocyte gene expression. Low versus high seizure frequency TLE is predicted by the respective overexpression and underexpression of specific systemic leukocyte DEGs involving neuroinflammation, oxidative stress and lipid peroxidation, GABA (γ-aminobutyric acid) inhibition, and AMPA (α- amino-3-hydroxy-5-methyl-4-isoxazolepropionic acid) and NMDA (N-Methyl- d-aspartate) receptor signaling. In addition, high seizure frequency-temporal lobe epilepsy is distinguished prognostically from low seizure frequency-temporal lobe epilepsy by differentially increased specific leukocyte gene expression involved in GABA (γ-aminobutyric acid) inhibition and NMDA (N- Methyl- d-aspartate) receptor signaling. An in-depth look at the upregulated leukocyte DEGs within their associated pathophysiological pathways is necessary to help provide a possible mechanistic view of these biomarkers with prognostic value for TLE seizure frequency. Here we discuss some of these DEGs in detail and their likely role in epileptogenesis in the context of known pathogenic signaling pathways.

### TLE epileptogenicity signaling pathways

**Fig. 8:**
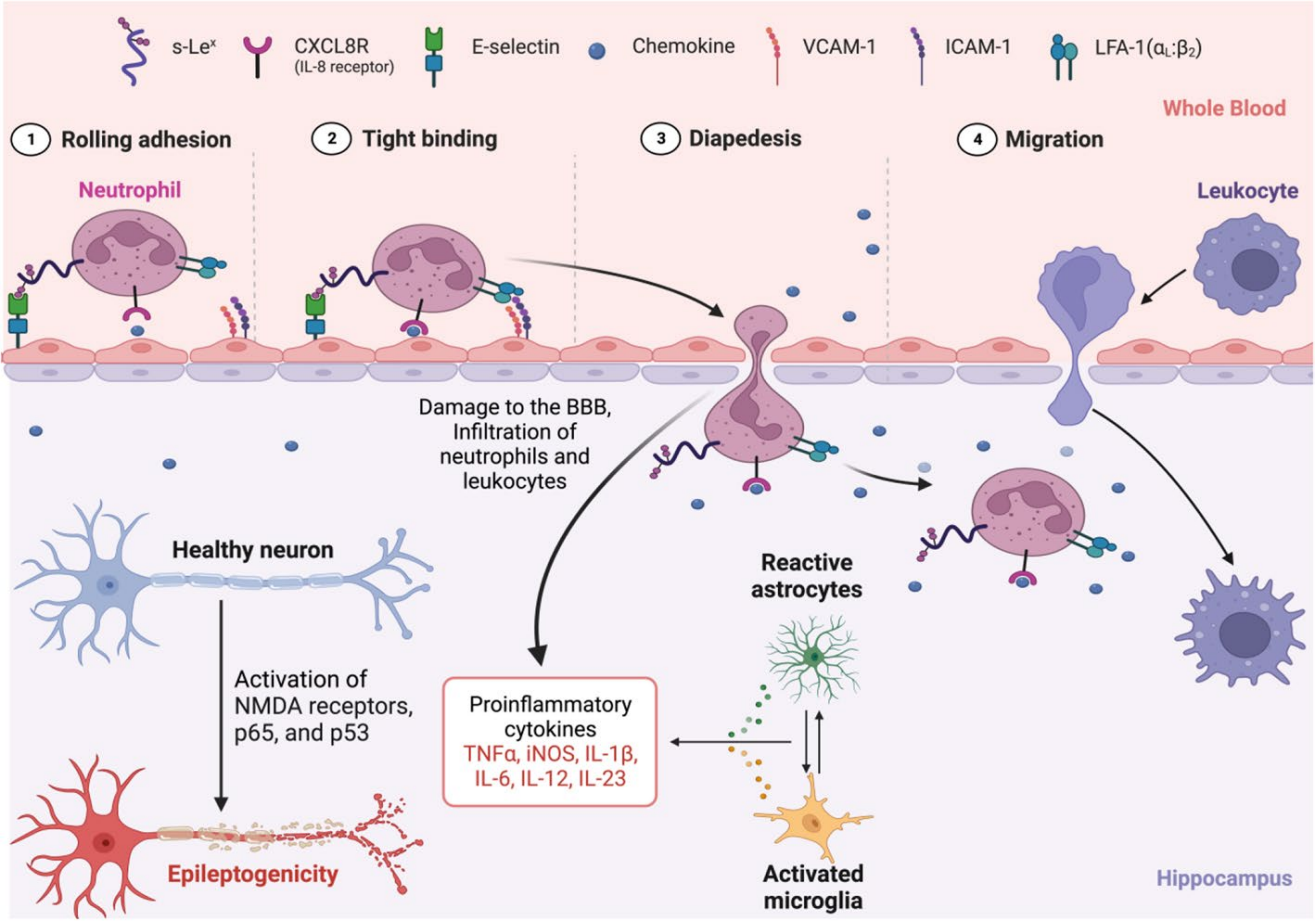
This schematic depicts the intricate process of leukocyte infiltration into the brain from the systemic bloodstream. Initially, the leukocytes engage with Vascular Adhesion Molecule 1 (VCAM-1) and Intercellular Adhesion Molecule 1 (ICAM-1), facilitating trans-endothelial diapedesis, a crucial event in the breaching of the blood–brain barrier (BBB). This breach contributes significantly to epilepsy-associated neuroinflammation. This process leads to the activation of pro-inflammatory cytokines, instigating a feed-forward cascade that further stimulates microglial cells. This exacerbated neuroinflammatory response augments epileptogenicity in patients with Temporal Lobe Epilepsy (TLE), implying a complex interplay between the immune response and the neurological disorder. Created with BioRender.com

The neurobiology of epilepsy has been characterized by the pathogenic “triad” of neuroinflammation, oxidative stress and glutamate-mediated excitotoxicity [19]. These pathophysiological mechanisms produce progressive neurodegeneration and recurrent seizures through network alterations, increased neuronal synchronization susceptibility and seizure- induced cell death [19]. Systemic leukocytes infiltrate the brain from the blood stream through VCAM-1 (Vascular Adhesion Molecule 1) and ICAM-1 (Intercellular Adhesion Molecule 1) adhesion molecules, trans-endothelial diapedesis, and blood–brain barrier disruption promoting epilepsy- associated neuroinflammation (Fig. 8) [8, 20]. Leukocytes perform an immunosurveillance function by trafficking through the brain, systemically recapitulating the pathophysiology of TLE, and express prognostic transcriptomic information in the treatment of TLE [8]. The current study demonstrates that systemic leukocyte gene expression has prognostic value differentiating low from high seizure frequency in refractory TLE. Among patients with temporal lobe epilepsy, low seizure frequency is prognostically differentiated from high seizure frequency by differentially increased specific leukocyte gene expression involved in the biological processes of neuroinflammation, oxidative stress and lipid peroxidation, and AMPA (α-amino-3-hydroxy-5-methyl-4-isoxazolepropionic acid) receptor signaling. High seizure frequency-temporal lobe epilepsy is distinguished prognostically from low seizure frequency-temporal lobe epilepsy by differentially increased specific leukocyte gene expression involved in GABA (γ-aminobutyric acid) inhibition and NMDA (N- Methyl- d-aspartate) receptor signaling. Additionally, there appears to be a larger scale suppression or enhancement of phagosome formation, CREB signaling and autophagy between the low and high seizure frequency cohorts that may also inform the mechanistic differences in these patients with TLE.

### Leukocyte gene expression predictive of decreased temporal lobe epilepsy seizure frequency

#### Neuroinflammation (RHOB, TLR1, TNFRSF1A, LYN, CASP1, PRKCD, FCGR2A, IFNGR1)

Neuroinflammation is known to promote temporal lobe epileptogenesis and epileptogenicity [21, 22]. In TLE, chronically activated systemic lymphocytes express inflammatory cytokines and traffic within the brain parenchyma exacerbating inflammation and neurotoxicity [8, 23, 24] (Fig. 3). Several leukocyte DEGs identified in this study are involved in neuroinflammation and their upregulation was identified when comparing low to high seizure frequency.

The small GTPase, RHOB (Ras Homolog Family Member B; Rho-Related GTP-Binding Protein RhoB), activates the dual transcription factors, NF-kB and STAT3, which are involved in (a) acute inflammation and (b) neural differentiation and immune response with a positive correlation with seizure frequency, respectively [25–27]. Specifically, among the classic RHO isoforms (RHOA, RHOB, RHOC), RHOB is uniquely endosomal and activates NF-kB, an acute inflammation transcription factor which is over-expressed in hippocampal CA1 and CA3 pyramidal neurons, reactive astrocytes and dentate granule cells and astrocytic processes in patients with medial temporal lobe epilepsy [28–30]. In glioblastoma cell lines, RHOB knockdown has been shown to cause decreased cytokine-induced STAT3 activation and impaired STAT3 activity [28]. Inflammatory pathways, including those involving STAT3, have been implicated in preclinical studies in the modulation and genesis of epilepsy after brain injury [31]. The JAK/STAT pathway specifically is activated in the rodent pilocarpine model of status epilepticus (SE), which produces temporal lobe epilepsy and the inhibition of STAT3-regulated gene transcription is shown to decrease long-term spontaneous seizure frequency following onset of status epilepticus [32]. In human epilepsy, serum levels of all STATs are elevated, with STAT3 being the most profound at a nine-fold increase above baseline [21].

Another gene identified in our study, PRKCD (PKCδ, Protein Kinase C Delta), is involved in the epilepsy inflammatory response and has been shown to increase neuronal excitability and epileptogenesis [33]. PKCδ is a proinflammatory, oxidative stress-inducing and epileptogenic factor in temporal lobe epilepsy and various isoforms of Protein Kinase C (PKC) are involved in epileptogenesis [34]. PKCδ is involved in the inflammatory response to epilepsy which is known to increase neuronal excitability, decrease the seizure threshold, enhance blood–brain barrier permeability and produce epiletognenesis [33]. PKCδ also activates several programmed cell death signaling pathways and hippocampal excitability in status epilepticus in rats [35]. PKCδ is the immediate downstream target of Fyn, a non-receptor Src family of tyrosine kinase (SFK) [36]. Hippocampal Fyn and PKCδ are increased following induction of the kainate model of status epilepticus and both Fyn and PKCδ demonstrate increased hippocampal microglial staining in epileptogenesis [36]. Similarly, in a transgenic model of murine temporal lobe epilepsy, knockdown of microglia PKCδ eliminates the microglial proinflammatory inflammogen-induced response and decreases release of proinflammatory mediators, TNF-α, IL-1β, IL-6, and IL-12, reducing electrographic non- convulsive seizure frequency, epileptiform spiking and neuronal degeneration [36].

Toll-like receptor 1 (TLR1) was also shown to be predictive of seizure frequency in our study and is implicated in temporal lobe epilepsy as a key transduction receptor of neuro- inflammation-induced epileptogenesis through glial production of inflammatory cytokines, IL-1β and TNF-α [37]. TLRs represent a 10-member family of transmembrane proteins which detect damage associated molecular patterns (DAMPs) and have been implicated as key signal transducers in neuro-degenerative disorders and neuroinflammation-induced epileptogenesis [38, 39]. Toll ligands promote neuronal and glial production of inflammatory cytokines including TNF-α, IL-1β**,** IL- 6 and other inflammatory mediators of epileptogenesis [38, 40, 41]. In patients with epilepsy, inflammation in brain tissue is predominately induced by the Toll-like receptors [37]. Also of note, epileptogenic tissue shows upregulated TLR1in neurons, microglia and astrocytes which mediates both adaptive and innate immune responses [37, 40]. Within this same pathway another gene detected in our study, the protein tyrosine kinase LYN (LYN Proto- Oncogene, Src Family Tyrosine Kinase), is known to activate toll-like receptors and regulates NF-kB while promoting over-expression of pro-inflammatory cytokines, IL-1β and TNF-α, accentuating neuronal hyperexcitability [42, 43]. Importantly, LYN is a known regulator of neuroinflammation, neuronal excitability, and epileptogenicity and is a member of the non- receptor Src protein tyrosine kinase (SFK) family [42]. SFKs are key signaling components of the immune response and microglial function and have been implicated in epileptogenesis [36, 42, 44]. During epileptogenesis, SFK upregulation in hippocampal microglia occurs concurrently with upregulation of pro-inflammatory cytokines, electrographic non-convulsive seizures and increased epileptiform spiking [36]. In contrast, in vitro inhibition of microglial LYN produces anti-inflammatory effects, including attenuated TNFα and IL-6 secretion [43]. Additionally, SFK inhibition decreases in vitro hippocampal epileptiform discharge frequency and in vivo duration and seizure numbers in mice [36, 45]. This correlation was also shown in our data.

Yet another gene detected in our study and known to be upregulated in human TLE is CASP1 (Caspase 1, Apoptosis-Related Cysteine Protease, Interleukin-1β Convertase). CASP1 is pro-inflammatory and pro-convulsant in human TLE and processes the major pro-inflammatory cytokine, Interleukin-1β (IL-1β), to an active secreted form, through leucine-rich repeat (LRR)- containing proteins (NLR) family member (NLRP) inflammasomes [46–48]. In temporal lobe epilepsy (TLE), inflammasomes are involved in the seizure-induced degenerative process in both animals and humans [49]. The NLRP1 inflammasome, expressed in both neurons and glial cells, exerts a crucial role in seizure-induced neuronal damage [50–52]. Inflammasomes activate CASP1 and the transcription factor, NFkB, which both activate the pro-inflammatory cytokine, IL-1β, promoting epileptogenicity [53, 54]. In human temporal lobe epilepsy patients, Caspase 1 is often upregulated in the hippocampi of patients with temporal lobe epilepsy and silencing of Caspase 1 or NLRP1 produces neuroprotective and antiepileptic effects which is also consistent with our data [49]. Similarly, FCGR2A (FcγRIIA, Fc Fragment of IgG Receptor IIa) can modulate pro- and anti-inflammatory signaling via receptors of the Fc (fragment crystallizable) region of immunoglobulins (FcRs) which bridge the cellular and humoral pathways of the immune system [55–57]. Specifically, FcγRIIA and FcγR mediate pro- inflammatory signaling pathways, neuro- and excitotoxicity, and lipid peroxidation which in turn promote epileptogenicity [55, 56, 58–60]. Interestingly, cultured cortical and hippocampal cells, exposed to IgG-IC, induce FcγR-mediated IgG internalization, Erk phosphorylation and increased intracellular calcium [58]. FcγR signaling is also responsive to the pro-inflammatory cytokine, IFNγ, and neuronal FcγRs contribute to kainic-acid brain neurotoxicity [58].

Lastly, the gene IFNGR1 (Interferon Gamma Receptor 1, IFN-γ R1), which is pro- convulsant through activation of TNFR1 and TLR1 inflammatory pathways, operates through the pro-inflammatory cytokine, interferon- γ (IFN- γ). IFN- γ is produced by T and natural killer (NK) cells and microglia and binds to the IFN-γ receptor consisting of IFN-γR1 and IFN-γR2 subunits [57, 61]. IFN-γR1 is expressed in both glial cells and neurons [62]. Th1 cells secrete IFN-γ inducing microglia into a pro-inflammatory, cytotoxic M1 phenotype [63]. In patients with temporal lobe epilepsy, peripheral lymphocytes are in a chronic state of activation and demonstrate increased IFN-γ expression compared to healthy controls [24]. In patients with epilepsy, post-ictal and interictal peripheral blood concentrations of IFN-γ are also elevated, relative to healthy controls, and interictal IFN-γ concentration is positively correlated with seizure frequency [21, 63].

The involvement of RHOB, TLR1, TNFRSF1A, LYN, CASP1, PRKCD, FCGR2A, and IFNGR1 in neuroinflammation promotes temporal lobe epileptogenicity (Fig. 3). Leukocyte expression of these neuroinflammation generating genes is uniquely activated in TLE patients with low seizure frequency when compared to patients with high seizure frequency in this study.

#### Oxidative stress (RAC2, NCF2, HMOX1)

Oxidative stress associated enzymes and markers are elevated in surgically resected human epileptogenic brain tissue and are involved in epileptogenesis [64, 65]. Upregulation of leukocyte NCF2, RAC2 and HMOX1 expression in this study is predictive of low temporal lobe epilepsy seizure frequency, while downregulation of these genes predicts high seizure frequency. All of these genes play a role in oxidative stress and can be implicated in the process of redox imbalance (Fig. 4).

RAC2 (Family Small GTPase 2) is an important neutrophil component of the NADPH oxidase (NOX) multi-protein complex and regulates generation of oxidative stress (OS) reactive oxidative species (ROS), superoxide (O_2_^−^) and hydrogen peroxide (H_2_O_2_), which are demonstrated in animal and in vitro models of epilepsy [64, 66]. NOX activation generates high levels of neuronal and glial lipid peroxidation in human epileptogenic CA1 neuronal hippocampal plasma membranes [66].

The NCF2 transcript protein, p67phox, is an important cytosolic component of the NADPH oxidase (NOX) multi-protein complex which works in concert with RAC2 to maximize electron transport [66]. Phagocyte NADPH oxidase regulates major immune system pathways including type 1 interferon signaling, inflammasomes and autophagy as well as the generation of oxidative stress (OS), reactive oxidative species (ROS), superoxide (O_2_^−^) and hydrogen peroxide (H_2_O_2_) [66]. There is emerging evidence that temporal lobe epilepsy is a disease of redox imbalance involving reactive oxygen species pathways [64, 67, 68]. As such, temporal lobe epilepsy ictal electrocorticographic and clinical seizure onset appear to be epiphenomena, preceded by more fundamental physiologic perturbations [64, 67, 68]. For instance, in the cat hippocampal penicillin model of epilepsy, Ammon’s horn neuronal mitochondrial redox changes precede electrographic ictal onset by over 3 min [67]. This pre-ictal decline in NADH is due to NADH oxidation and suggests that hippocampal neuronal energy change contributes to epileptogenesis [67]. Similarly, HMOX1 upregulation is also associated with mitochondrial oxidative stress, but also with NF-κB-associated neuronal toxicity and glial-related neurodegeneration [69]. HMOX1 is known to be a ubiquitous and redox-sensitive inducible stress protein [65]. Additionally, mitochondrial astrocytic integrity is undermined by HMOX1 which mediates gliopathy, lowering the threshold of neighboring neuronal elements to oxidative injury [70]. In cultured rat astroglia, miRNAs, regulated by HMOX1, cause mitochondria-dependent apoptosis and cell death, enhance TNFα biosynthesis which may worsen bioenergetic insufficiency, and compromise neural oxidative phosphorylation [70]. HMOX1 dysregulation is also significant in its role of iron-induced production of free radicals [71]. Iron generates free radicals which damage cell membranes via lipid peroxidation producing neuronal injury, increased extracellular glutamate and excitation as well as decreased GABA-A receptor function and inhibition, and epileptic discharges [72]. In a rat model of cortical and neuronal hemin toxicity, neuronal cell death is prevented by treatment with the anticonvulsant, Valproic acid, which inhibits hemin toxicity by downregulation of HMOX1 [73]. This is further evidenced by the pentylenetetrazole (PTZ) kindled mouse model of epilepsy which produces significant neuronal cell loss associated with oxidative stress, lipid peroxidation and enhanced hippocampal HMOX1 expression [74].

Heme Oxygenase 1 (HMOX1) catabolizes heme to carbon monoxide, biliverdin and free ferrous iron (Fe2 +), the latter inducing production of free radicals, lipid peroxidation and oxidative stress producing neuronal injury and epileptic discharges [71, 72]. Receptors of the Fc (fragment crystallizable) region of immunoglobulins (FcRs), including FcγRIIA, regulate antibody-dependent immune-complex-triggered inflammation through superoxide anions, lipoprotein oxidation and neurotoxicity and are associated with immune complex-mediated seizures [59, 60]. The hippocampal microglia Src protein tyrosine kinase (SFK), LYN, upregulates nitro-oxidative stressors with production of electrographic seizures and increased epileptiform spiking [75].

Enzymes involved in oxidative stress and oxidative stress markers have been shown to be elevated in surgically resected human epileptogenic brain tissue, supporting a redox imbalance hypothesis of human temporal lobe epileptogenesis [64]. Also, oxidative stress activates inflammasomes which are important drivers of inflammation, neuronal degeneration, and neurodegenerative disease, including epilepsy [76]. NCF2, RAC2 and HMOX1 activity all promote epileptogenicity through mitochondrial oxidative stress, neuronal lipid peroxidation, and neuronal and glial toxicity, all of which are consistent with the redox imbalance hypothesis of temporal lobe epilepsy (Fig. 4).

#### Glutamate/GABA-mediated excitotoxicity (LYN, MYD88, CHP1, TNFRSF1A)

Glutamate is the main excitatory neurotransmitter in the brain and, through ionotropic glutamate receptors, controls most excitatory neurotransmission [77]. N-methyl-D-aspartate (NMDA) and 2-amino-3-(3-hydroxy-5-methyl-isoxazol-4-yl) propanoic acid (AMPA) ionotropic glutamate receptors (a) mediate neuronal hyperexcitability and are present in human epilepsy and in kindling models of epilepsy and (b) initiate epileptiform discharges, mediate fast synaptic excitation, and are important in the spread and synchronization of epileptic activity, respectively [36, 77–79]. Patients with temporal lobe epilepsy demonstrate reduced GABAergic- associated inhibition within the epileptic hippocampus and decreased numbers of GABA-ergic interneurons and GABA-ergic terminals and absent functional inhibition contributing to spontaneous recurrent seizures [80, 81] (Fig. 5).

As a Src protein tyrosine kinase (SFK), another gene found in this study, LYN.

(LYN Proto-Oncogene, Src Family Tyrosine Kinase), upregulates NMDA receptor mediated neuronal hyperexcitability [43, 45, 82]. These SFKs upregulate NMDA receptor (NMDAR) mediated neuronal hyperexcitability through phosphorylation and augmentation of NMDARs [36, 82, 83]. LYN activation of P2X4 receptors produces microglial brain-derived neurotrophic factor (BDNF) secretion increasing NMDAR currents affecting neuronal plasticity and excitability [42, 44]. Conversely SFK inhibition decreases in vitro hippocampal epileptiform discharge frequency and in vivo duration and number of seizures in mice [36, 45]. Also in the NMDR signaling pathway is another gene found in our study, MYD88 (MYD88 Innate Immune Signal Transduction Adaptor). MYD88 is an intracellular adapter protein involved in the IL-1β signaling cascade which causes increased Tyr 1472-phosphorylation of the NR2B subunit of the NMDA receptor (NMDAR) and pharmacologic inhibition of MYD88 prevents the proconvulsant effects of IL-1β [84, 85]. Inhibition of hippocampal MYD88 reduces N-methyl-D-aspartate receptor NR1 subunit expression and increases glutamate transporter 1 expression, protecting pyramidal neurons from apoptosis [86]. Interestingly, MYD88 may also be responsible for NMDA receptor expression [86].

Another gene seen in our study related to Glutamate/GABA-Mediated Excitotoxicity is CHP1. CHP1 (calcineurin homologous protein 1) upregulation is shown to inhibit NHE1 (Na^+^/H^+^ exchanger). Loss of the NHE1 (Na^+^/H^+^ exchanger 1) isoform in turn inhibits GABA-loaded vesicle release and causes central nervous system hyperexcitability ultimately resulting in epilepsy [87, 88]. CHP subfamily targeting of NHEs is complex, depending on the applied stimulus and cellular environment; activity of the NHE1 isoform may be accentuated or inhibited by binding to CHP1 [89]. NHE1, the principal target of CHP1, confers resistance to apoptosis and loss of NHE1, a neuronal plasma membrane constituent of the hippocampus and cortex, increases central nervous system excitability and produces epilepsy [87, 88, 90]. Mice which lack NHE1 also demonstrate increased hippocampal neuronal excitability and higher Na^+^ channel subtype I current density [89, 91].

Lastly, the gene TNFRSF1A, in addition to being pro-inflammatory, also activates AMPA signaling creating pro-convulsant conditions. Specifically, TNFα alters the molecular stoichiometry and modulates the homeostatic synaptic scaling of post-synaptic AMPARs (AMPA (alpha-amino-3-hydroxy-5-methyl- 4-isoxazolepropionic acid receptors)-glutamate receptors), increases hippocampal neuronal cell surface GluA-AMPARs and increases excitatory synaptic activity of TNFRSF1A receptors [92–94]. Post-synaptic AMPAR down-scaling and decreased overall AMPAR scaling capacity have been suggested as explanations for maintaining high and low seizure thresholds in temporal lobe epilepsy patients with low and high seizure frequency, respectively [12].

### Leukocyte gene expression predictive of increased temporal lobe epilepsy seizure frequency

#### Biological pathways involved in increased temporal lobe epilepsy seizure frequency (AK1, F2R, GNB5, TYMS)

Increased temporal lobe epilepsy seizure frequency is predicted by specific leukocyte gene expression involving activation of NMDA and inhibition of GABAergic signaling (Fig. 6). F2R mechanisms induce NMDA receptor potentiation and inflammatory signaling pathways, while TYMS protein production leads to homocysteine (Hcy) accumulation facilitating NMDA signaling. AK1 associated AMP production, accentuated AMPK signaling, and microglial mTOR inhibition and GNB5 activity inhibit neuronal GABAergic and GABA_B_ R-GIRK signaling, respectively. The combined NMDA receptor facilitation and GABAergic inhibition together produce the increased epileptogenicity responsible for increased temporal lobe seizure frequency (Fig. 6).

One of the genes detected in these pathways is Adenylate kinase 1 (AK1). As a key enzyme in adenine nucleotide metabolism, AK1 catalyzes the reaction of ATP + AMP ↔ 2ADP, producing extra energy under metabolic stress [95, 96]. Temporal lobe epilepsy is a disease of energy metabolism, with the interictal epileptic temporal lobe demonstrating reduced glucose utilization [97]. AK1 is also highly expressed in the brain and plays important roles in degenerative diseases involving inflammation, hypoxic conditions and oxidative stress [95, 98]. AK1 knockout mice demonstrate reduced cardiac tolerance to ischemic stress, impaired nucleotide salvage during re-perfusion, and decreased contraction-mediated AMPK (AMP-activated protein kinase) phosphorylation consistent with decreased AMP production [99]. Critically, AMPK is an upstream inhibitor of mTOR (mechanistic or mammalian target of rapamycin) signaling; this is important as inhibition of microglial mTOR produces neuronal loss and increased spontaneous seizures [100–102]. Inhibited of mTOR signaling may reduce sprouting of somatostatin- positive interneurons with inhibitory synapses of GABAergic neurons in the hilus [103]. The predictive value of increased leukocyte AK1 expression for high TLE seizure frequency is consistent with AK1 associated AMP production, accentuated AMPK signaling, microglial mTOR and GABAergic neuronal inhibition, and enhanced epileptogenicity (Fig. 6).

Another gene identified as having increased expression in the high seizure frequency cohort is Coagulation Factor II Thrombin Receptor (F2R), also known as Protease-Activated Receptor 1 (PAR1). This is the primary thrombin receptor in the brain [104]. PARs are G-protein coupled receptors responsible for thrombosis, hemostasis, inflammatory response, apoptosis, cell growth and proliferation [105, 106]. Thrombin levels increase significantly following status epilepticus (SE) [104]. Through neuronal and glial cell PAR activation, increased thrombin may produce inflammation, apoptosis and neuronal excitability through NMDA receptor potentiation which then produces glutamate-induced excitotoxicity and neurodegeneration [104, 106, 107]. F2R significantly induces inflammation through COX-2 expression and Prostaglandin E2 release and initiates in vivo and in vitro signaling cascades for neuronal and glial p38 and p42/44 MAPK, mitogen-activated protein kinases (MAPKs), including ERK1/2, c-Jun N-terminal kinase, and Rhoa/Rho kinase, and protein kinase B which are up-regulated in the brains of intractable epilepsy patients [106]. F2R activation also causes increased neuronal and non-neuronal intracellular Ca2 + concentration and produces an increase in membrane depolarization, inducing and amplifying seizures [106]. It has also been shown that the lack of or inhibition of F2R confers significant neuroprotection [106]. Inhibition of F2R is neuroprotective, anti-epileptic, reduces SE (status epilepticus)-induced cell loss, rescues SE-induced hippocampal CA1 region synaptic plasticity, increases hippocampal neuronal cell survival, suppresses interictal spikes and decreases behavioral seizures after SE, decreases mortality, and decreases the occurrence of epilepsy in experimental temporal lobe epilepsy [104, 106, 108]. This F2R-mediated signaling is shown to be involved in SE-induced epileptogenesis and has been suggested as a potential molecular target for antiseizure drug therapy [106]. The finding that increased leukocyte F2R gene expression predicts increased temporal lobe epilepsy seizure frequency is also consistent with known F2R mechanisms which induce NMDA receptor potentiation and inflammatory signaling pathways involved in epilepsy (Fig. 6).

The next gene identified to be predictive of high seizure frequency is GNB5 (G Protein Subunit Beta 5). GNB5 encodes guanine nucleotide‐binding protein sub-unit beta‐5 (Gβ5) which downregulates central nervous system G-protein signaling through interactions with G-protein receptors [109]. Homozygous and compound heterozygous GNB5 mutations result in a multisystem syndrome with cardiac conduction, and ocular and neurological disorders, including epilepsy [109]. G-protein–activated inward-rectifying K + (GIRK) channels assemble with Gβ5 and control neuronal excitability, specifically by inhibiting synaptic transmission via neuronal hyperpolarization [110]. GABA_B_R stimulation in the hippocampus is mediated by G protein-gated inwardly-rectifying K^+^ (GIRK/Kir3) channels [111]. G protein signaling proteins negatively modulate GABA_B_R-GIRK signaling [111]. Absence of Gβ5 produces dramatic slowing of GIRK channel deactivation kinetics with prolongation of synaptically-evoked slow inhibitory post-synaptic currents (IPSCs) and slowing of hippocampal GABA_B_R-GIRK response deactivation rates [111, 112]. Gβ5 proteins also modulate GABA_B_R signaling via deactivation of GIRK channels on hippocampal neuronal post-synaptic dendritic spines and inhibit hippocampal pyramidal neuronal GABA_B_R-GIRK signaling [113]. Increased leukocyte GBN5 gene expression is again prognostic for increased temporal lobe epilepsy seizure frequency and consistent with the mechanism of GBN5 inhibition of GABA_B_R-GIRK signaling and increased epileptogenicity (Fig. 6).

Lastly, Thymidylate Synthetase (TYMS), a key gene involved in DNA damage repair, was also shown to be upregulated in the high seizure frequency cohort [114]. Increased levels of TYMS protein leads to homocysteine (Hcy) accumulation [114]. Hcy is proconvulsant and produces intraneuronal fibrillar amyloid beta conformation, promoting neurodegeneration [115]. In the pilocarpine rodent model of temporal lobe epilepsy, the proposed mechanism for Hcy enhancement of seizure induction is a synergistic, facilitatory activation of NMDA receptor signaling through the NMDA receptor allosteric modulatory site [115]. Increased Hcy levels are frequently associated with anticonvulsant therapy [115]. Hcy is taken up by neurons, reduces the seizure threshold and increases seizure frequency in patients treated with anticonvulsant medication [115]. Increased TYMS leukocyte gene expression is prognostic for high seizure frequency temporal lobe epilepsy and also fits with the mechanism of increased homocysteine protein levels, facilitation of NMDA signaling and increased epileptogenicity (Fig. 6).

#### Top altered canonical pathways inferred from pathway enrichment analysis

Lastly, we wanted to use IPA to look at the top altered canonical pathways to see if there were distinctions between the low and high seizure frequency cohorts leukocyte expression profiles. This analysis showed consistent patterns of suppression or enhancement of phagosome formation, neuroinflammation signaling, CREB signaling, and autophagy between the low and high frequency seizure groups (Table 6).

Carmona-Mora et al. [116] performed RNAseq analysis on isolated neutrophils, monocytes and whole blood from patients that experienced intracerebral hemorrhage (ICH) and ischemic stroke (IS) to better understand the immune response to brain injury. Monocyte genes were down- regulated in both IS and ICH, whereas neutrophil gene expression changes were generally up- regulated. We found that several of the pathways in Table 6 were shared with those enriched in the ICH monocyte data of Carmona-Mora et al. [116], including neuroinflammation [117], Recognition of bacteria and viruses [118], TREM-1 signaling [119] and IL-8 signaling [120]. All of these pathways were deactivated in monocytes from ICH, as was TREM-1 signaling in monocytes from IS patients. The most significantly over-represented suppressed pathways in IS monocytes included phagosome formation, as well as interferon signaling and dendritic cell maturation. None of the pathways in Table 6 were shared with the neutrophilic pathways in Carmona-Moras et al. [116], or vice versa.

Communication between injured ischemic brain and peripheral blood leukocytes drives changes of the peripheral immune system that affect stroke pathophysiology and outcome [121].

Monocytes (which account for ~ 2–8% of peripheral leukocytes) change phenotypes in response to their microenvironment, differentiating into different subtypes and to macrophages [122]. If our results do indeed support deactivation of these pathways in HSF patients, this may indicate a link with processes that occur in the blood of stroke patients. Interestingly, similar to Carmona- Mora et al. [116], the top predicted upstream deactivator inferred by IPA was interferon (IFNγ) (Fig. 7); Carmona-Mora et al. [116] found that the interferon pathway was suppressed in monocytes and suggested that this could occur by down-regulation of pro-inflammatory pathways, such as TREM1 signaling. In addition, IFNγ has been associated with stroke-induced neurodegeneration since it is pro-inflammatory [123]. The down-regulation of these upstream regulators and pro-inflammatory pathways may suggest peripheral monocytes switch to more anti-inflammatory functions in HSF patients.

The top pathway in Table 6, phagosome formation, is an integral part of the process of autophagy, as are several of the other pathways listed in Table 6 [124]. Autophagy is a catabolic process by which proteins and organelles are delivered to the lysosome for degradation, and functions as a survival mechanism that maintains cellular homeostasis under normal growth conditions and enables adaptation under stress [124]. Autophagy is initiated by the formation of a unique membrane structure, the phagophore, which engulfs part of the cytoplasm and forms a double-membrane vesicle termed the autophagosome. Fusion of the outer autophagosomal membrane with the lysosome and degradation of the inner membrane contents complete the process.

Interestingly, we find 3.4-fold lower expression of the v-ATPase gene (ATP6AP2) in leukocytes of HSF compared with those of LSF patients (*p*-value = 8.40 × 10^–5^, FDR = 1.78 × 10^–2^). ATP6AP2 has recently been found to be identical to the (pro)renin receptor and has a role in the renin- angiotensin system that also regulates V-ATPase activity. The vacuolar-type H + -ATPases (V- ATPases), which are ATP-driven proton pumps responsible for modulating intracellular and extracellular pH, are involved in many physiological processes, including autophagy and apoptosis. The transmembrane fragment of Atp6ap2 contributes to v-ATPase function, which plays an important role in autophagy. Phagocytic pathways require acidification of the (phago-)lysosomes which is established by v-ATPase [125]. Lysosomes are needed to degrade macromolecules from different pathways such as endocytosis, autophagy and phagocytosis [126]. Therefore it is possible that down-regulation of v-ATPase (ATP6AP2) interferes with autolysosome formation by blocking autophagic flux, the dynamic process of autophagy [127]. We also found that the autophagy-related gene, ATG3, is expressed at 3.1-fold lower levels in HSF versus LSF (*p*-value 3.09 × 10^–6^, FDR = 1.93 × 10^–3^). ATG3 is one of the key genes involved in autophagy, and its homologs are common in eukaryotes. During autophagy, ATG3 acts as an E2 ubiquitin-like conjugating enzyme in the ATG8 conjugation system, contributing to phagophore elongation [124].

#### Limitations

This study aimed to identify specific leukocyte gene expression parameters that can effectively differentiate TLE patients into two distinct groups based on seizure frequency: high and low. However, the absence of a control group, comprising healthy individuals or patients with different forms of epilepsy, hampers the assessment of gene expression differences between TLE patients with low and high seizure frequencies, as well as healthy controls. Furthermore, the study had a small sample size, including 16 patients. This limited sample size may compromise the generalizability of the findings and provide a less comprehensive understanding of the complexities associated with temporal lobe epilepsy. This study was not able to assess the time to most recent seizure relative to the blood sample collection which may confound the gene expression results of the study.

## Conclusions

Systemic leukocyte gene expression has prognostic value for human temporal lobe epilepsy (TLE) seizure frequency, with thirteen differentially expressed leukocyte genes identified that differentiate low from high TLE seizure frequency. It's observed that low human TLE seizure frequency is predicted by upregulation and high TLE seizure frequency is predicted by downregulation of leukocyte expression of thirteen specific genes involved in the temporal lobe epileptogenicity “triad” of (a) neuroinflammation, (b) oxidative stress and lipid peroxidation, and (c) glutamate/GABA-mediated excitotoxicity. Moreover, high TLE seizure frequency is predicted by increased expression and low TLE seizure frequency is predicted by decreased expression of four additional, specific leukocyte genes involved in NMDA and GABA-mediated epileptogenicity. Leukocytes are thought to perform an immunosurveillance function by trafficking through the brain, systemically recapitulating the pathophysiology of TLE, and expressing prognostic transcriptomic information with relevance for the treatment of TLE. Given the dynamic nature of temporal lobe epilepsy, it could be informative to investigate changes in leukocyte gene expression over time. Longitudinal studies that include multiple time points during the disease history could provide insights into the dynamics of gene expression and its correlation with seizure frequency. Finally, leukocyte gene expression seems to dichotomize TLE into two distinct entities, with unique implications for pathophysiology and potential disease therapy, based on low and high seizure frequency. This suggests that low and high seizure frequency patients may represent two mechanistically different forms of temporal lobe epilepsy based on leukocyte gene expression.

## Data Availability

All Fastq files and gene expression data have been deposited into NCBI's Gene Expression Omnibus (GEO) repository. Accession# GSE217726 - Leukocyte gene expression predicts human temporal lobe epilepsy seizure frequency.

## Abbreviations

TLE: Temporal lobe epilepsy
DEGs: Differentially expressed genes
FDR: False Discovery Rate
FC: Fold change
IPA: Ingenuity Pathway Analysis
AMPA: α-Amino-3-hydroxy-5-methyl-4-isoxazolepropionic acid
NMDA: N-Methyl-d-aspartate
GABA: γ-Aminobutyric acid
ROS: Reactive oxygen species
SFKs: Src family of tyrosine kinases
SE: Status epilepticus
NLRP: NLR family member
IL-1β: Interleukin-1β
IFN-γ: Interferon gamma
FcRs: Fc receptors
ICAM-1: Intercellular Adhesion Molecule 1
VCAM-1: Vascular cell adhesion protein 1
s-Le^x^: Sialyl LewisX
CXCL8R: Interleukin 8
LFA-1: Lymphocyte function-associated antigen 1
PET: Positron emission tomography
EEG: Electroencephalogram
MRI: Magnetic resonance imaging
MTS: Medial temporal sclerosis
AH: Amygdalohippocampectomy
ATL: Anterior temporal lobectomy
SLAH: Stereotactic laser amygdalohippocampotomy
ACEP: Arizona Comprehensive Epilepsy Program
RNA-Seq: RNA sequencing
GEO: Gene Expression Omnibus
HSF: High seizure frequency
LSF: Low seizure frequency
